# A Presumed Acquired Ureteral Diverticulum Presenting With Hematuria, Urosepsis, and Periureteral Contrast Leak: A Rare Case Report

**DOI:** 10.7759/cureus.115534

**Published:** 2026-08-31

**Authors:** Tariq Abdul Hamid, Fadhel Yusuf, Ammar Agha, Hussain A Al Sadi, Murtadha Altammam, Shaima M Abuhejleh, Mohamad Hasan, Hamzeh Esmaeilpour, Fariborz Bagheri

**Affiliations:** 1 Surgery, Dubai Hospital, Dubai Health, Dubai, ARE; 2 Urology, Dubai Hospital, Dubai Health, Dubai, ARE; 3 Medicine, Dubai Hospital, Dubai Health, Dubai, ARE

**Keywords:** acquired ureteral diverticulum, conservative management, hematuria, periureteric contrast leak, retrograde pyelography, ureteral diverticulum, ureteroscopy, urosepsis

## Abstract

Ureteral diverticulum is an exceptionally rare abnormality that may be congenital or acquired secondary to chronic obstruction, calculi, infection, or prior instrumentation. While often asymptomatic, complicated cases can present with hematuria, recurrent UTIs, or sepsis.

A 60-year-old bedridden woman with morbid obesity and a history of multiple endourological procedures presented with urosepsis and hematuria. Computed tomography demonstrated mild hydroureteronephrosis and a periureteric fluid collection with gas locules and contrast extravasation, initially raising suspicion of an infected urinoma. Subsequent retrograde pyelography and rigid ureteroscopy confirmed a narrow-necked ureteral diverticulum at the level of the sacroiliac joint. Following antibiotic therapy and urinary drainage, the patient improved clinically and was managed conservatively due to her high operative risk, remaining asymptomatic at follow-up.

Acquired ureteral diverticulum should be considered in patients with hematuria, urosepsis, and a history of prior endourological procedures when cross-sectional imaging shows periureteric collections or contrast leakage. Retrograde pyelography and ureteroscopy are essential for distinguishing a diverticulum from free ureteral perforation. Conservative management with urinary drainage is a viable approach in selected high-risk patients.

## Introduction

Ureteral diverticulum is a rare urologic entity defined as a localized saccular outpouching arising from the ureteral wall. Despite being described in older urologic literature, it remains uncommon, with fewer than 50 well-documented cases summarized in a contemporary review of the literature [1]. Early classification work helped distinguish true ureteral diverticula from related developmental anomalies and provided the foundation for differentiating congenital lesions from acquired diverticula [2]. Later etiologic studies proposed that chronic obstruction, increased intraluminal pressure, infection, and ureteral wall weakness may contribute to diverticulum formation [3,4]. Congenital diverticula have been described in both children and adults, with radiologic and clinical variability depending on size, location, and associated urinary tract abnormalities [5]. Acquired diverticula have also been reported after ureteral reconstructive surgery, supporting the role of iatrogenic ureteral wall alteration in selected cases [6].

The clinical spectrum is broad. Many ureteral diverticula are asymptomatic and detected incidentally, whereas symptomatic cases may present with hematuria, recurrent UTIs, flank pain, obstruction, urinary stasis, or stone formation within the diverticular cavity [7]. Imaging is central to diagnosis, and sonography may identify a cystic periureteric lesion in selected patients, although demonstration of communication with the ureter is often required for confirmation [8]. Antenatally detected proximal ureteral diverticulum has also been reported, highlighting that some lesions may have a congenital origin and may be recognized early in life [9]. Large diverticula can mimic pelvic cysts or other cystic retroperitoneal collections, making preoperative diagnosis challenging [10]. When intervention is required, reported options include laparoscopic excision, robotic resection, segmental ureteral reconstruction, or other procedures tailored to diverticulum location, renal function, and patient factors [11,12]. More recent adult case reports continue to emphasize that retrograde pyelography, CT urography, and endoscopic assessment are useful when cross-sectional imaging is equivocal [13-15]. Rarely, ureteral diverticula or diverticulum-like abnormalities have been described in association with urothelial carcinoma or benign fibroepithelial polyps, making recurrent hematuria or suspicious mucosal changes clinically important during follow-up [16-18].

Ureteral diverticula are usually classified into three main categories: abortive ureteral duplications, true congenital diverticula containing all layers of the ureteral wall, and acquired diverticula caused by mucosal herniation through a weakened ureteral wall [1,2,5]. Acquired diverticula are thought to develop in association with chronic or intermittent elevation of intraluminal pressure, ureteral obstruction, calculi, infection, previous ureteral surgery, or repeated instrumentation [1,3,4,6]. This mechanism is clinically relevant because patients with recurrent stone disease or multiple endourological procedures may develop ureteral wall inflammation, stricturing, localized weakness, and subsequent outpouching over time.

We present a rare case of presumed acquired ureteral diverticulum in a 60-year-old bedridden woman with chronic catheterization, recurrent right-sided stone disease, and multiple previous endourological procedures. The patient presented with hematuria, urosepsis, and imaging evidence of a periureteric collection with contrast leakage. The diagnosis was confirmed by retrograde pyelography and ureteroscopy, and the patient was successfully managed conservatively because of her high surgical risk.

This work was previously presented as a poster at the Interdisciplinary Urology Care Consortium (IUCC) 2025.

## Case presentation

A 60-year-old bedridden woman with morbid obesity presented to the ED with several days of visible hematuria. Her baseline immobility was related to a previous road traffic accident. She had a chronic indwelling urethral catheter and a history of multiple endourological procedures over the preceding five years for right ureteric and renal calculi. Her indwelling urethral catheter had recently been changed uneventfully at another facility.

On arrival, she was febrile and hypotensive, with a temperature of 39°C, blood pressure of 80/50 mmHg, heart rate of 110 beats per minute, and oxygen saturation of 95% on 2 L of oxygen via nasal cannula. Initial laboratory evaluation demonstrated severe leukocytosis, profound elevation of inflammatory markers, and acute kidney injury consistent with urosepsis and hemodynamic compromise (Table 1).

**Table 1:** Laboratory investigations on admission.

Laboratory parameter	Patient value	Standard reference range
WBC count	27 × 10⁹/L	4.0-11.0 × 10⁹/L
CRP	148 mg/L	<5.0 mg/L
Procalcitonin (PCT)	37.9 ng/mL	<0.15 ng/mL
Serum creatinine	1.58 mg/dL	0.50-1.10 mg/dL
N-terminal pro-B-type natriuretic peptide (NT-proBNP)	3,101 pg/mL	<125 pg/mL

Electrocardiography showed atrial fibrillation with a rapid ventricular response, with a heart rate of 111 beats/minute. She received an IV amiodarone 300 mg bolus followed by a maintenance infusion, after which sinus rhythm was restored (Figure 1). Therapeutic enoxaparin at 1 mg/kg twice daily was initiated during the evaluation for thromboembolic disease, and CT pulmonary angiography later excluded pulmonary embolism.

**Figure 1:**
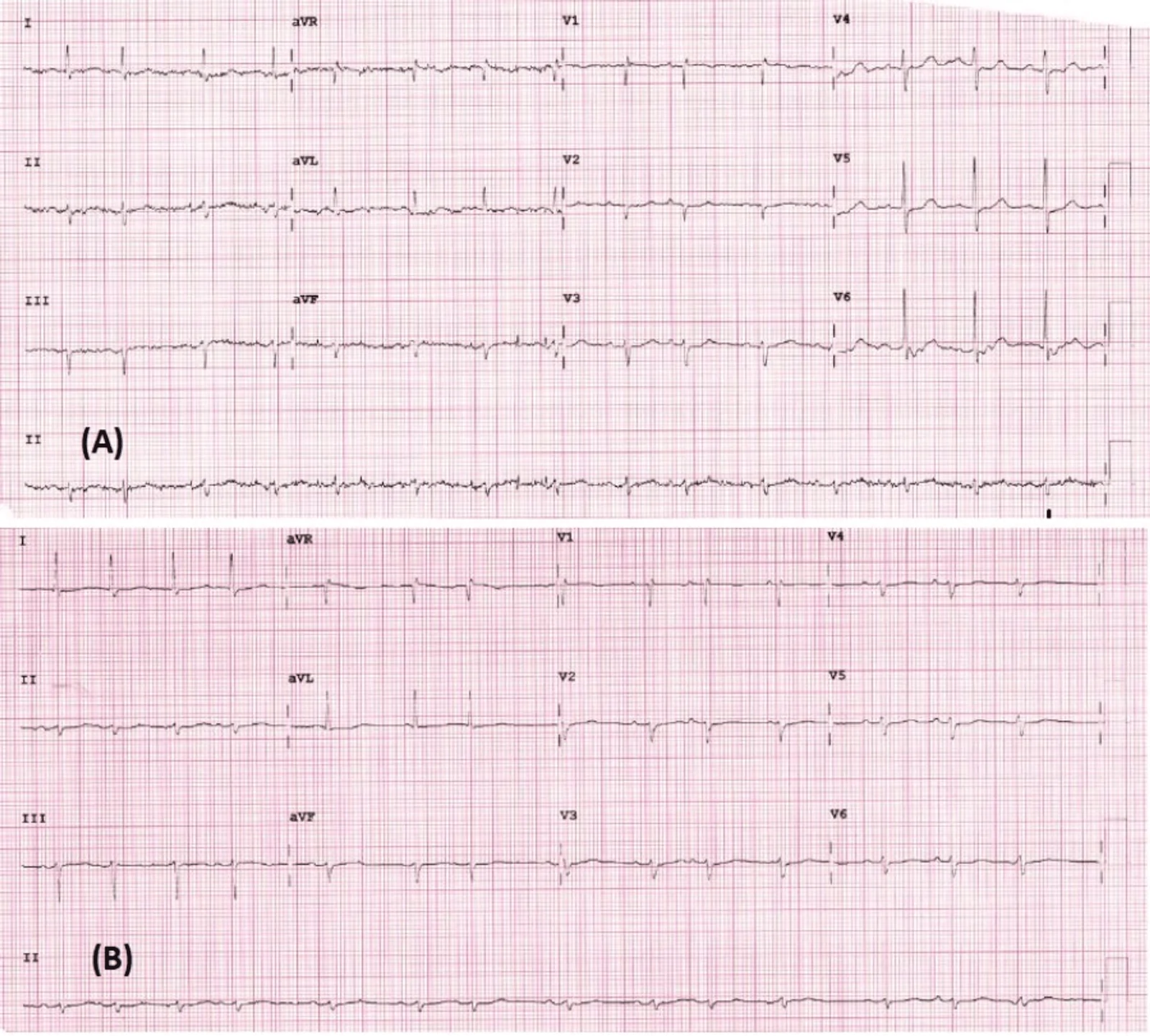
(A) Twelve-lead ECG demonstrating atrial fibrillation, characterized by the absence of discrete P waves, irregularly irregular R-R intervals, and a variable ventricular response. (B) Twelve-lead ECG showing normal sinus rhythm, with clearly identifiable P waves preceding each QRS complex and regular R-R intervals.

Contrast-enhanced CT of the abdomen and pelvis (Figure 2) demonstrated a malrotated atrophic right kidney with mild right hydroureteronephrosis and a periureteric fluid collection measuring approximately 7.3 × 3.0 × 6.8 cm surrounding the proximal to mid-right ureter on delayed-phase images. The collection contained tiny gas locules with surrounding inflammatory fat stranding. Based on these findings, the initial radiologic impression favored an infected urinoma or localized periureteric collection secondary to ureteric contrast extravasation rather than a ureteral diverticulum, which was considered only after subsequent endoscopic evaluation.

**Figure 2:**
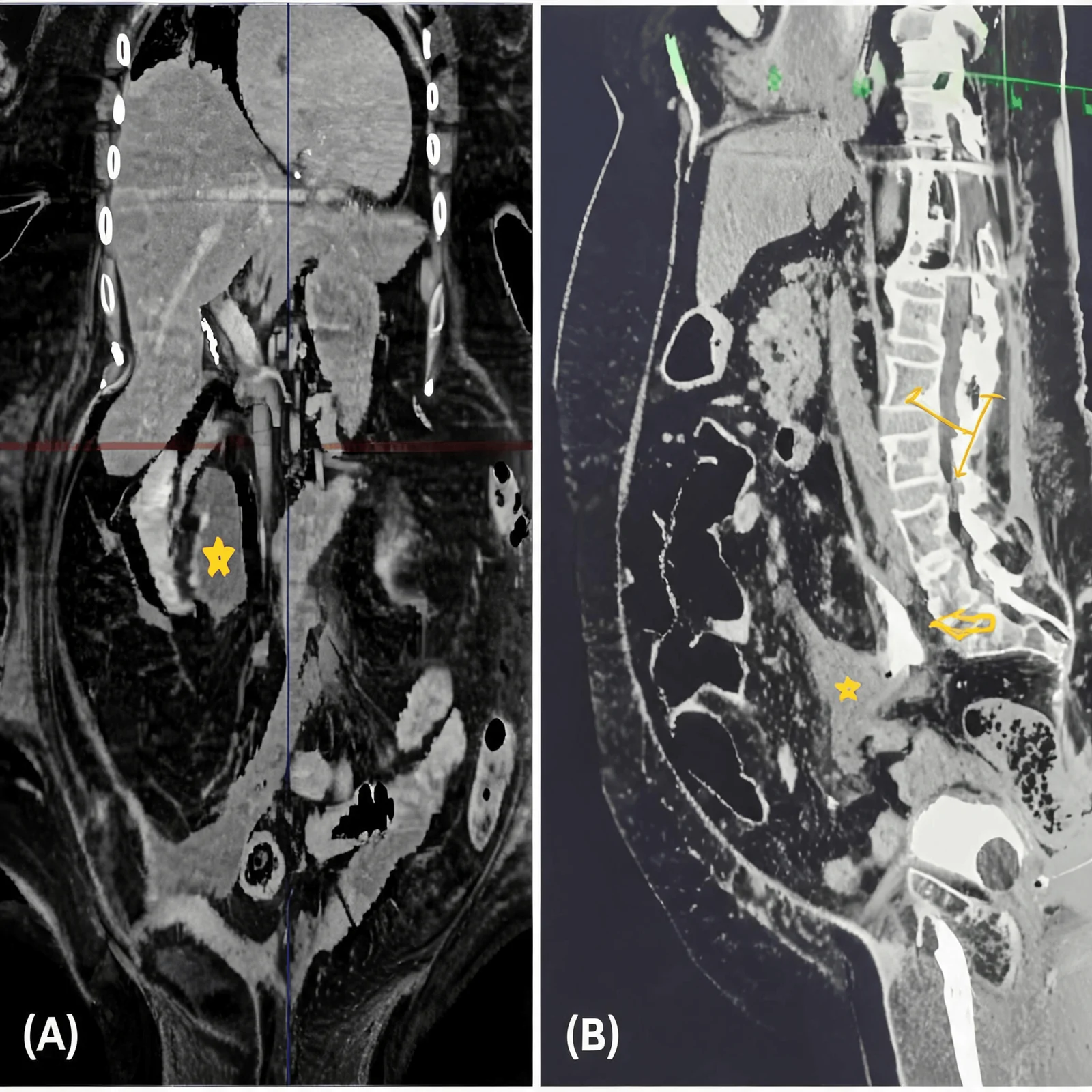
Contrast-enhanced computed tomography (CECT) of the abdomen and pelvis. (A) Coronal view demonstrating a malrotated, atrophic right kidney with mild right hydroureteronephrosis and a periureteric fluid collection surrounding the right ureter. The yellow star denotes the fluid collection. (B) Sagittal view showing contrast extravasation on delayed films from the mid-to-proximal right ureter (yellow arrow) opening into a walled-off periureteric collection at the level of the sacroiliac joint (yellow star).

The patient underwent cystoscopy and retrograde evaluation. Cystoscopy revealed a markedly reduced bladder capacity of approximately 50 mL, with no abnormal bladder lesions or masses. Retrograde pyelography demonstrated a confined contrast-filled collection at the level of the sacroiliac joint, corresponding to the periureteric abnormality seen on CT (Figure 3A).

**Figure 3:**
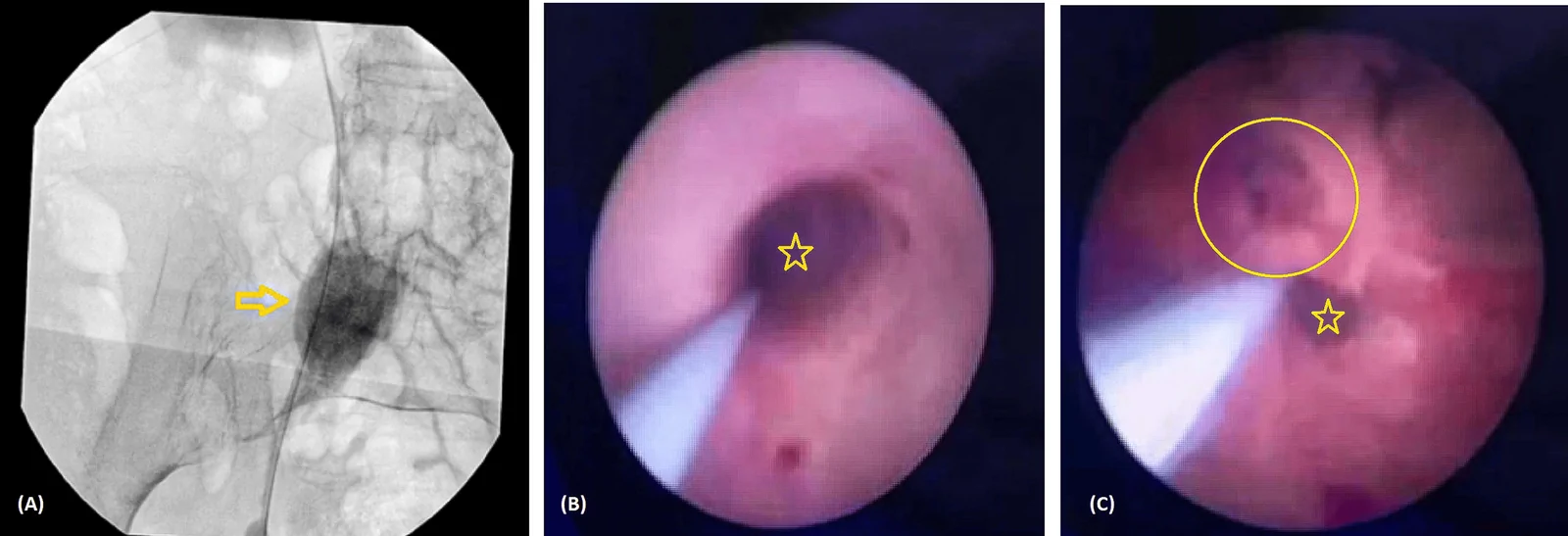
Endoscopic and retrograde radiologic confirmation of the diverticulum. (A) Retrograde pyelography demonstrating opacification of a well-confined periureteric cavity at the level of the sacroiliac joint (arrow). (B and C) Rigid ureteroscopy views showing the narrow neck of the ureteral diverticulum, with edematous but benign-appearing mucosa and no abnormal lesions or masses (circle), as well as the adjacent healthy proximal ureteral lumen (star).

Rigid ureteroscopy demonstrated a narrow-necked ureteral diverticulum communicating with the ureteral lumen. The diverticular mucosa appeared mildly edematous without evidence of calculi, papillary lesions, suspicious masses, or other abnormal mucosal changes. The adjacent proximal ureter appeared healthy without evidence of ureteric perforation or stricture, confirming that the contrast-filled cavity represented a ureteral diverticulum rather than free ureteral extravasation (Figures 3B-3C).

A ureteric catheter and an 18 Fr silicone Foley catheter were inserted to provide urinary drainage. The patient was treated with IV meropenem 1000 mg every 8 hours. Her post-procedural course was stable, with marked clinical and laboratory improvement. Blood and urine cultures, which were obtained after the administration of empirical antibiotics, were negative, and no further episodes of hematuria occurred during admission.

Because of the patient’s bedridden status, morbid obesity, comorbid frailty, and high operative risk, definitive surgical excision was deferred. A conservative management plan was established, including serial clinical assessment, renal function monitoring, repeat imaging as needed, catheter care, and regular urology follow-up. At the 3-month follow-up, the patient remained clinically stable with no recurrence of hematuria or urosepsis.

## Discussion

Ureteral diverticulum remains an unusual diagnosis, and the distinction between congenital and acquired forms is important. True congenital diverticula contain the normal layers of the ureteral wall, whereas acquired diverticula are generally considered mucosal herniations through a weakened ureteral wall and may lack a complete muscular layer [1,2,5]. Abortive ureteral duplication, sometimes described as a blind-ending branch of a bifid ureter, represents a separate developmental category [1,2]. In the present case, the diagnosis was most consistent with an acquired diverticulum because the patient had a long history of ipsilateral stone disease, repeated endourological procedures, chronic urinary catheterization, and an atrophic malrotated kidney with mild hydroureteronephrosis. However, because the diverticulum was not surgically excised, its acquired nature was inferred from the clinical context rather than confirmed histologically.

The pathogenesis of acquired ureteral diverticulum is not fully established. Proposed mechanisms include chronic obstruction, increased ureteral intraluminal pressure, recurrent infection, stone-related inflammation, ureteral wall dyskinesia, and iatrogenic weakening after surgery or instrumentation [1,3,4,6]. Older etiologic studies and later case reports support the concept that abnormal hydrodynamic stress across a weakened ureteral segment can lead to progressive outpouching [3,4]. Acquired diverticula have also been reported after reconstructive ureteral surgery, emphasizing that surgical alteration of ureteral anatomy can predispose to focal diverticular formation [6]. In our patient, recurrent calculi and repeated ureteroscopic interventions may have contributed to chronic ureteral wall injury and localized structural weakness.

Clinical presentation is variable. Many patients are asymptomatic, and the abnormality is discovered incidentally on imaging or during retrograde studies [1,13,15]. Symptomatic diverticula may cause hematuria, recurrent UTI, pyelonephritis, flank pain, urinary stasis, stone formation, obstruction, or renal functional impairment [1,7,13,14]. Rarely, large diverticula may resemble pelvic cysts, abscesses, urinomas, or other cystic retroperitoneal collections [10]. This diagnostic overlap was distinctly evident in our case. The initial cross-sectional imaging features, specifically a periureteric fluid collection accompanied by local gas locules and significant fat stranding, strongly pointed toward an infected urinoma secondary to an acute ureteral perforation. This demonstrates how severe localized inflammation can obscure the structural communication between a diverticulum and the ureteral lumen on a standard CT scan. Ultimately, controlled retrograde pyelography and direct ureteroscopic visualization ruled out free perforation or an abscess and established the diagnosis of a contained, narrow-necked ureteral diverticulum.

The imaging approach to suspected ureteral diverticulum should be individualized. CT is useful in acutely ill patients because it can assess obstruction, renal morphology, periureteric inflammation, extravasation, abscess formation, and alternative causes of hematuria or sepsis. CT urography or delayed excretory-phase imaging may demonstrate communication between the ureter and a diverticular sac, but the sensitivity of cross-sectional imaging can be limited when the cavity is poorly opacified or when inflammation, infection, or gas locules obscure the anatomy [1,13,15]. USG may detect cystic periureteric lesions but is less definitive unless communication with the ureter is demonstrated [8]. Retrograde pyelography remains highly valuable because controlled contrast injection can distend and opacify the diverticulum, allowing direct visualization of its communication with the ureter [1,13]. In this case, retrograde pyelography was the key investigation that converted a suspected leak or urinoma into a defined ureteral diverticulum.

Endoscopic assessment adds diagnostic and therapeutic value. Ureteroscopy can directly identify the diverticular opening, evaluate the health of the adjacent ureteral lumen, exclude obstructing stones, and assess for suspicious mucosal lesions. This is relevant because chronic urinary stasis, recurrent inflammation, and diverticular anatomy may complicate follow-up, and rare reports have described urothelial carcinoma or benign fibroepithelial polyps arising in association with ureteral diverticula or diverticulum-like abnormalities [16-18]. Although urothelial carcinoma has been reported in association with ureteral diverticula, endoscopic assessment in the present case demonstrated only mildly edematous mucosa without papillary lesions, masses, or other suspicious mucosal abnormalities. Therefore, no endoscopic features suggestive of malignancy were identified. Nevertheless, continued surveillance remains appropriate should recurrent hematuria or new radiologic abnormalities develop.

Management depends on symptoms, complications, renal function, and patient fitness for surgery. Uncomplicated and asymptomatic ureteral diverticula are often managed conservatively with observation [1,3]. Intervention is generally reserved for complicated cases, including recurrent infection, persistent hematuria, obstruction, stone formation, progressive hydronephrosis, functional deterioration, suspected malignancy, or failure of conservative therapy [1,11,12,14]. Reported surgical options include diverticulectomy, segmental ureteral resection with ureteroureterostomy, ureteral reimplantation for distal disease, nephroureterectomy or nephrectomy in poorly functioning renal units, and minimally invasive laparoscopic or robotic approaches [11,12].

Endoscopic assessment adds diagnostic and therapeutic value. Ureteroscopy can directly identify the diverticular opening, evaluate the health of the adjacent ureteral lumen, exclude obstructing stones, and assess for suspicious mucosal lesions. This is relevant because chronic urinary stasis, recurrent inflammation, and diverticular anatomy may complicate follow-up, and rare reports have described urothelial carcinoma or benign fibroepithelial polyps arising in association with ureteral diverticula or diverticulum-like abnormalities [16-18]. Although urothelial carcinoma has been reported in association with ureteral diverticula, endoscopic assessment in the present case demonstrated only mildly edematous mucosa without papillary lesions, masses, or other suspicious mucosal abnormalities. Therefore, no endoscopic features suggestive of malignancy were identified. Nevertheless, continued surveillance remains appropriate should recurrent hematuria or new radiologic abnormalities develop.

Management depends on symptoms, complications, renal function, and patient fitness for surgery. Uncomplicated and asymptomatic ureteral diverticula are often managed conservatively with observation [1,3]. Intervention is generally reserved for complicated cases, including recurrent infection, persistent hematuria, obstruction, stone formation, progressive hydronephrosis, functional deterioration, suspected malignancy, or failure of conservative therapy [1,11,12,14]. Reported surgical options include diverticulectomy, segmental ureteral resection with ureteroureterostomy, ureteral reimplantation for distal disease, nephroureterectomy or nephrectomy in poorly functioning renal units, and minimally invasive laparoscopic or robotic approaches [11,12].

In the present case, the immediate priorities were sepsis control, urinary drainage, and stabilization. Ureteric catheter drainage, Foley catheterization, and broad-spectrum intravenous antibiotics led to rapid clinical and biochemical improvement, and hematuria resolved without further intervention. Although internal ureteral stenting was considered in our case, a temporary ureteric catheter was selected because endoscopic evaluation demonstrated no evidence of ureteric obstruction, stricture, or ongoing perforation requiring prolonged internal drainage. In addition, the patient's multiple comorbidities, frailty, and poor functional status would have made future stent exchanges technically challenging and associated with additional anesthetic risk. This management strategy achieved adequate urinary drainage while avoiding the need for repeated interventions. Conservative management was therefore appropriate, provided that she remained under close surveillance for recurrent infection, recurrent hematuria, obstruction, progressive hydronephrosis, or renal functional decline.

This case adds to the limited literature by illustrating a presumed acquired ureteral diverticulum presenting not merely as an incidental finding but with hematuria, urosepsis, and radiological suspicion of periureteric contrast leakage. It also highlights the diagnostic importance of retrograde pyelography and ureteroscopy in distinguishing a contained diverticular cavity from free ureteral perforation, an infected urinoma, or an abscess.

## Conclusions

Ureteral diverticulum is rare but should be considered in patients with hematuria, recurrent UTIs, prior stone disease, and a history of ureteral instrumentation, particularly when CT demonstrates a periureteric collection or suspected contrast leak. Retrograde pyelography and ureteroscopy are valuable confirmatory investigations when cross-sectional imaging is inconclusive. This case also highlights that inflammatory periureteric collections initially interpreted as a urinoma or ureteric contrast leak on CT may ultimately represent a contained ureteral diverticulum, emphasizing the diagnostic value of retrograde pyelography and ureteroscopy. In selected high-risk patients who improve after urinary drainage and antibiotic therapy, conservative management with structured follow-up may be a safe and reasonable approach.

## References

[1] McLoughlin LC, Davis NF, Dowling C, Eng MP, Power RE (2013). Ureteral diverticulum: a review of the current literature. Can J Urol.

[2] Culp OS (1947). Ureteral diverticulum classification of the literature and report of an authentic case. J Urol.

[3] Barrett DM, Malek RS (1975). Ureteral diverticulum. J Urol.

[4] Rank WB, Mellinger GT, Spiro E (1960). Ureteral diverticula: etiologic considerations. J Urol.

[5] Sarajlić M, Durst-Zivković B, Svoren E, Vitković M, Batinić D, Bradić I, Vucković I (1989). Congenital ureteric diverticula in children and adults: classification, radiological and clinical features. Br J Radiol.

[6] Aragona F, Bassi P, Passerini GG, Pagano F (1989). Acquired intravesical ureteral diverticulum: an unusual late complication of ureteroneocystostomy. J Urol.

[7] Mori C, Yamada D, Homma Y (2011). A case of calculus in the true ureteral diverticulum. Int J Urol.

[8] Wan YL, Hsieh ML, Hsueh C, Shum HC, Kea CL (1996). Sonographic diagnosis of a ureteral diverticulum. J Ultrasound Med.

[9] Herndon CD, McKenna PH (2000). Antenatally detected proximal ureteral diverticulum. Urology.

[10] Slavkovic A, Vacic N, Jovanovic Z, Zivkovic V (2011). Gigantic ureteral diverticulum or pelvic cyst?. J Pediatr Surg.

[11] Li JR, Chiu KY, Lin HM, Cheng CL, Yang CR, Hung SW (2006). Laparoscopic excision of a ureteral diverticulum. Int J Urol.

[12] Mehra K, Sreerag KS, Manikandan R, Dorairajan LN, Kalra S, Avinash J (2020). Rare entity of proximal ureteral diverticulum managed by robotic resection and ureteroureteral anastomosis. J Endourol Case Rep.

[13] Abdullatif V, Consolo M (2021). Ureteral diverticulum in a 65-year-old female. Cureus.

[14] Mejri R, Chaker K, Mokhtar B, Ben Rhouma S, Nouira Y (2021). Ureteral diverticulum complicated by urinary lihiasis: about a case report. Urol Case Rep.

[15] Emekli E, Gündoğdu E, Özen A, Küçükay F (2021). A rare case of ureteral diverticulum incidentally detected during angiography. Curr Med Imaging.

[16] Harrison GS (1983). Transitional cell carcinoma in a congenital ureteral diverticulum. J Urol.

[17] Parker MD, Rebsamen S, Clark RL (1989). Multiple ureteral diverticula: a possible radiographically demonstrable risk factor in development of transitional cell carcinoma. Urol Radiol.

[18] Franco I, Choudhury M, Eshghi M, Bhalodi A, Addonizio JC (1988). Fibroepithelial polyp associated with congenital ureteral diverticulum: report of 2 cases. J Urol.

